# Targeted delivery of PGE_2 _ameliorates autoimmune nephritis

**DOI:** 10.1186/ar4620

**Published:** 2014-09-18

**Authors:** Nino Kvirkvelia, Maggie McMenaminn, Michael P Madaio

**Affiliations:** 1 Georgia Regents University, Augusta, GA, USA

## Background

In SLE, pathogenic mechanisms responsible for immune activation, immune deposit formation, cell infiltration and organ damage are complex and variable. However, immune cells and their products contribute to the nature and intensity of organ involvement as well as the host's response to therapy. In many respects, renal involvement is a microcosm of systemic disease, with variable glomerulonephritis, interstitial nephritis and vasculitis. Both systemic autoimmune and renal cellular responses influence individual disease phenotype. Nevertheless, antigenic specificity determines local events, in particular where immune deposits form. Direct binding of autoantibodies to glomerular antigens is particularly relevant, and the site of complex formation, along with the capacity of deposited Ig to engage FcR and activate complement play important roles in disease expression. Cellular infiltration (for example, T cells, B cells, macrophages) and the renal cellular response to these events are also important and influential. For the most part, therapy is systemic and directed at controlling inflammation and autoimmunity, rationalizing that limiting autoreactivity and inflammatory cell infiltration will control disease. Although this approach is somewhat efficacious, it has limitations. Thus, we reasoned that in some circumstances, targeted therapy could provide advantage of better ongoing disease in a given organ, while sparing systemic side effects.

## Methods

To approach this problem, we postulated that human monoclonal anti-α3(IV) antibodies (Ab) that localize in glomeruli could serve as vehicles for targeted drug delivery for glomerular diseases, including lupus nephritis. As a potential disease-modifying agent, we took advantage of recent observations that PGE_2 _enhanced renal cellular recovery and regeneration after established immunologic injury during the course of nephrotoxic nephritis (NTN). To enhance efficacy and limit undesirable systemic effects and target the kidney, PGE_2 _was coupled to a human monoclonal anti-α3(IV) Ab. Given enhanced glomerular expression of α3(IV), with very limited epitope exposure in other organs, it provides an ideal target for delivery of glomerular disease modifying agents. Therefore, PGE_2 _was chemically linked to human anti-α3(IV) Ab. Chemical composition of conjugates was assessed by western blotting, and preserved anti-α3(IV) activity was confirmed by ELISA.

## Results

Initially, glomerular localization of the anti-α3(IV) Ab-PGE_2 _conjugates was determined after injection in normal mice (IF). Once confirmed, the capacity of the conjugates to modify disease was determined during established NTN. Proteinuria, BUN levels and histology normalized in PGE_2 _conjugate-treated mice, as compared with untreated mice or mice treated with an equivalent dose of PGE_2 _alone.

## Conclusions

The results provide a novel means of targeting glomeruli during systemic disease, by providing efficient drug delivery, while limiting systemic effects.

